# Incidence, risk and impact of ICU readmission on patient outcomes and resource utilisation in tertiary level ICUs in Nepal: A cohort study

**DOI:** 10.12688/wellcomeopenres.18381.2

**Published:** 2023-02-28

**Authors:** Diptesh Aryal, Hem Raj Paneru, Sabin Koirala, Sushil Khanal, Subhash Prasad Acharya, Arjun Karki, Dilanthi Gamaga Dona, Rashan Haniffa, Abi Beane, Jorge I F Salluh

**Affiliations:** 1 Nepal Intensive Care Research Foundation, Kathmandu, Nepal; 2 IDOR - D'Or Institute for Research and Education, Rio de Janeiro, Brazil; 3 Pulmonology and Critical Care, Hospital for Advanced Medicine and Surgery (HAMS), Kathmandu, Nepal; 4 Mahidol Oxford Tropical Medicine Research Unit, Faculty of Tropical Medicine, Mahidol University, Bangkok, Thailand; 5 Critical Care Medicine, Tribhuvan University Teaching Hospital, Kathmandu, Nepal; 6 Critical Care Medicine, Grande International Hospital, Kathmanu, Nepal; 7 National Intensive Care Surveillance - MORU, Colombo, Sri Lanka; 8 Centre for Inflammation Research, University of Edinburgh, Edinburgh, UK; 9 Post Graduation Program, Federal University of Rio de Janeiro, Rio de Janeiro, Brazil

**Keywords:** ICU readmission; Incidence; Risk factor; Registry; LMICs

## Abstract

**Background**: Readmissions to Intensive Care Units (ICUs) result in increased morbidity, mortality, and ICU resource utilisation (e.g. prolonged mechanical ventilation), and as such, is a widely utilised metric of quality of critical care. Most of the evidence on incidence, characteristics, associated risk factors and attributable outcomes of readmission to ICU are from studies performed in high-income countries This study explores the determinants of risk attributable to ICU readmission in four ICUs in Kathmandu, Nepal.

**Methods**: The registry-embedded eCRF reported data on case mix, severity of illness, in-ICU interventions (including organ support), ICU outcome, and readmission characteristics. Data were captured in all adult patients admitted between September 2019 and February 2021. Population and ICU encounter characteristics were compared between those with and without readmission. Independent risk factors for readmission were assessed using univariate analysis.

**Results**: In total 2955 patients were included in the study. Absolute ICU readmission rate was 5.69 % (n=168) for all four ICUs. Median time from ICU discharge to readmission was 3 days (IQR=8,1). Of those readmitted, 29.17% (n=49) were discharged at night following their index admission. ICU mortality was higher following readmission to ICU(p=0.016) and mortality was increased further in patients whose primary index discharge was at night(p= 0.019). Primary diagnosis, age, and use of organ support in the first 24hrs of index admission were all independently attributable risk factors for readmission.

**Conclusions**: ICU readmission rates were adversely associated with significantly poorer outcomes, increased ICU resource utilisation. Clinical and organisational characteristics influenced risk of readmission and outcome.

## Abbreviations

**Table:** 

CCAA	Collaboration for Research, Implementation and Training in Asia-Africa
HIC	High Income Country
ICU	Intensive Care Unit
LMIC	Low and Lower Middle Income Country
NICRF	Nepal Intensive Care Research Foundation
RRT	Renal replacement therapy
UK	United Kingdom
US	United States

## Introduction

Readmissions to Intensive Care Units (ICU) result in increased morbidity, mortality, and ICU resource utilisation (e.g. prolonged mechanical ventilation), and as such, is a widely utilised metric of quality of critical care ^1, 2^.

ICU readmission is defined as admission to intensive care unit of a patient previously admitted to the ICU (index ICU admission) during the same hospital encounter ^1^. Early ICU readmissions are those occurring within 48 hours of ICU discharge; whilst ICU readmission occurring 3 to 14 days after discharge (during the same hospital encounter) is defined as late readmission ^3, 4^. Considering the detrimental impacts of readmission within 48 hours for both patients and critical care services, research in UK, US and European health systems has in recent years focused on increasing understanding of the determinants for risk of early readmission and in developing interventions to prevent avoidable harms. Attributable risks of early ICU readmission include individual patients’ characteristics, care processes in the ICU and organisational factors both within the ICU and in the wider hospital ^5– 7^. While individual patients’ characteristics are not necessarily modifiable, understanding the determinants associated with readmission is key to early recognition of at-risk population and the potential to modify the care pathway accordingly. The identification of organisational factors (e.g communication between healthcare teams, staffing ratios and variations in skills mix during daytime versus night/weekend) provides opportunities to adapt existing ICU discharge practices to mitigate their impact.

The majority of the evidence on incidence, characteristics, associated risk factors and attributable outcomes of readmission to ICU are from high-income country critical care populations and health systems (HICs) ^1, 8– 10^. The impact of ICU readmissions may be greatest in resource constrained health care systems (where patient outcomes may already be worse that predicted), however evidence regarding burden, character and factors which drive re-admission in such settings remain largely absent ^11, 12^. Furthermore the impact of organisational factors on outcomes, and strategies to mitigate and reduce avoidable readmission may or may not be translatable between health systems with differing resource availability. Multi-centre clinical quality registries increasingly established internationally, may provide a valuable source of data on case-mix, care processes and organisational factors and outcomes of critically ill patients. This study describes the incidence, impact and factors associated with increased risk of ICU readmissions in four ICUs in Nepal.

## Methods

This was a multi-center retrospective observational cohort study including four tertiary care hospitals all situated in the urban greater Kathmandu area; three private teaching hospitals, and one government teaching hospital. NICRF is supported by and a founding member of Wellcome Collaboration for Research, Implementation and Training in Asia-Africa; a nine country network aimed to improve outcomes for critically ill patients by establishing a community of practices in Asia ^13, 14^. NICRF uses near real-time high-quality data captured through a cloud-based registry platform, which captures near real-time, high-quality data on case mix, outcomes and care quality metric, enabling setting prioritised improvement, training and research for critical care in Nepal ^15^. Critical care is a growing independent speciality in Nepal, but ICU bed availability in the countries is estimated to be 2.8 per 100,000 population, with 85% ICUs being provided by the private sector ^16^.

**Figure 1.**
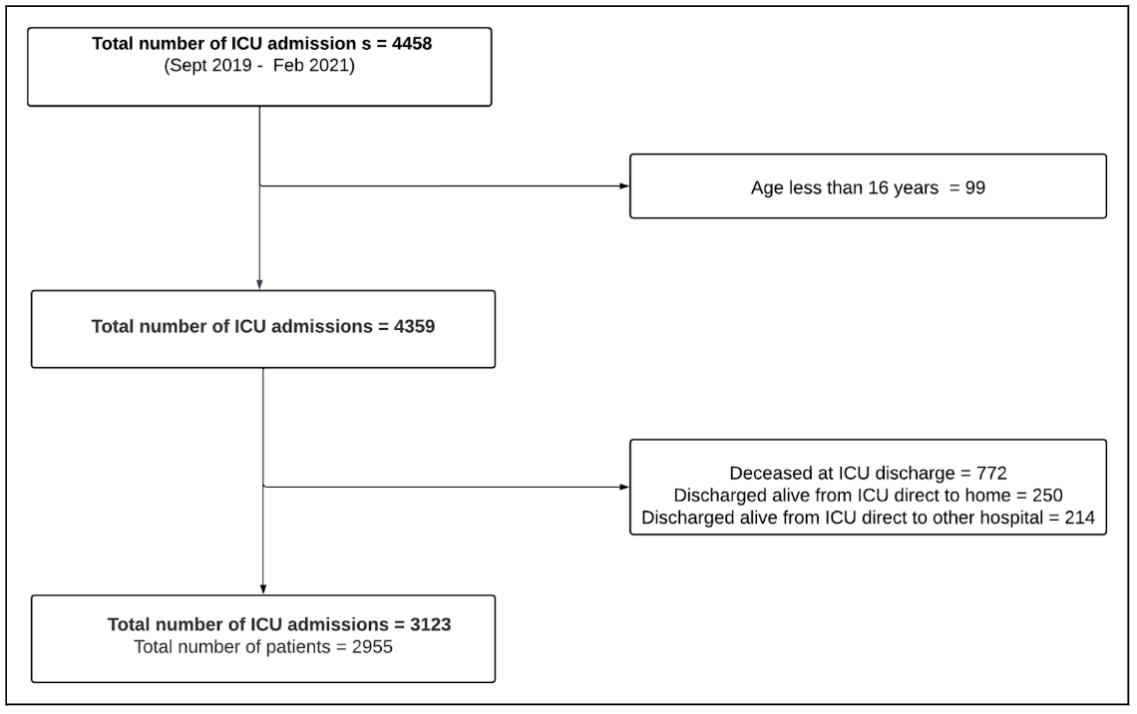
Flowchart of patients.

This study uses data captured routinely as part of the NICRF core data set. All consecutive ICU admissions from September 2019 to February 2021 were included and followed up until ICU discharge. Currently, the hospital discharge information remains an optional datapoint in the NICRF registry dataset. A flowchart of the study population is provided in Figure 1. The demographic, case mix and treatment characteristics for the ICU populations are described in additional file 2 ^17^. APACHE II was used to measure the severity of illness by calculating the most deranged reading during each patient's initial 24 h in ICUs ^18^. Patients who died in the ICU and those who were discharged alive but were, *a priori*, not considered suitable for ICU readmission, and those who were directly discharged from the ICU to home or another hospital were excluded from the analysis ^19^. Diagnosis was reported using APACHE IV classifications ^20^. Reason for index admission, severity of illness, duration of organ support, time of ICU discharge (prior to readmission) and length of stay for the index ICU admission were considered as potential predictors of readmission, based on existing literature ^5^. The primary outcome measured was the rate of ICU readmission. Readmission was defined as discharge to an area that provided a lower level of care followed by return to the same or a different ICU in the same hospital during the same hospital admission ^1^. The secondary outcomes recorded after each patient’s first ICU admission included in-ICU mortality and (where available) all-cause in-hospital mortality, ICU length of stay (LoS) for both the index ICU stay and following readmission, hospital discharge, and hospital LoS (where available). For each readmission we also recorded the time interval between initial ICU discharge and readmission. We further reported whether readmission was at night (from seven pm of the day to seven am next day) or a weekend (seven pm Friday to seven am Sunday). These time frames reflect current clinical working patterns and ‘weekend’ period in hospital-based healthcare in Nepal.

ICU readmission rates were calculated by dividing the number of readmissions by the number of patients who were discharged alive and eligible for ICU readmission ^9^. Readmissions were categorised as either early (occurring within 48hrs of ICU discharge following index admission) or late (occurring after 48 hours following ICU discharge). Continuous variables were presented as mean ± standard deviation or median (25–75% interquartile range, IQR), as appropriate and categorical variables were presented as absolute and relative frequencies. Normality was assessed by the Kolmogorov Smirnov test ^21^. Comparisons were made between patients with and without readmission to the ICU, between readmission which occurred at night time and day time, and between readmission within 48 hours or less and after 48 hours after the ICU discharge. Categorical variables were compared with chi-square test or Fisher exact when appropriate ^22^. Continuous variables were compared using independent *t*-test or Mann-Whitney U test in case of non-normal distribution ^23^. Univariate logistic regression analysis was performed to identify which predictors were associated with ICU readmission. Multivariate logistic regression analyses with backward elimination procedure including all predictors showing a *p* value <= 0.20 in the univariate analysis were undertaken to define which variables were independently associated with ICU readmission ^24^. We tested the linearity assumption for continuous variables included in the logistic regression model and the multicollinearity for the resultant model. Statistical tests were two-sided; a P value <0.05 was considered statistically significant. All the analyses were performed using STATA/IC for Mac v16.1 (StataCorp LP, Texas, USA). The analyses could be also be carried out using R Studio.

### Ethics approval

The study was approved by Nepal Health Research Council (Reference Number: 36012022 P).

Due to the retrospective nature of the study and as the analysis used anonymous registry data, the need for informed consent was waived by the ethical review board, Nepal Health Research Council.

## Results

During the study period 2955 ICU patients were eligible for inclusion ( Figure 1). Patient characteristics of the whole eligible population are described in detail in Table 1. The ICU readmission rate was 5.69 % (n=168) for all ICUs combined. The patients with medical diagnoses at index admission had a higher ICU readmission rate compared to the surgical patients (87.50% vs. 12.50%, p=0.016). Median time from ICU discharge to readmission was 3 days (IQR=8,1) and 39.88% of readmissions occurred within 48 hrs of their primary ICU discharge. Among the readmitted patients, 29.17% were discharged at night following their index admission. The readmitted population was older than their non-re admitted counterparts (61.5 (IQR=74,40.5) vs. 56 (IQR=69,39) years, p = 0.014).

**Table 1.** Comparison of index admission characteristics between patients with vs without readmission.

Characteristic	Total discharged patients eligible for readmission n=2955	Patients without readmission n=2787 (94.31%)	Patients with readmission, n=168 (5.69%)	P- value
Age in years, median (Q1-Q3)	56(39-69)	56(39-69)	61.5(40.5-74)	0.014 *
**Gender** Male Female Intersex	1869(63.25) 1084(36.68) 2(0.07)	1771(63.55) 1014(36.38) 2(0.07)	98(58.33) 70(41.67) 0(0)	0.367
**Route to admission** Non-operative Postoperative	2375(80.37) 580(19.63)	2228(79.94) 559(20.06)	147(87.50) 21(12.50)	0.016 *
**Comorbidities (Top 10)** Hypertension Diabetes Type 2 Diabetes Cardiovascular diseases Hypothyroidism Renal failure, Moderate to severe Chronic pulmonary disease Respiratory disease, Severe Respiratory disease, Mild Other neurological condition	1019(34.48) 433(14.65) 165(5.58) 106(3.59) 86(2.91) 62(2.10) 44(1.49) 33(1.12) 35(1.18) 21(0.71)	957(34.34) 402(14.42) 158(5.67) 99(3.55) 80(2.87) 55(1.97) 40(1.44) 29(1.04) 33(1.18) 20(0.72)	62(36.90) 31(18.45) 7(4.17) 7(4.17) 6(3.57) 7(4.17) 4(2.38) 4(2.38) 2(1.19) 1(0.60)	0.497 0.152 0.410 0.677 0.600 0.054 0.326 0.108 0.994 0.854
**Non-Operative admission** **diagnosis** Cardiovascular Gastrointestinal Genitourinary Haematology Metabolic/Endocrine Musculoskeletal/Skin Neurologic Respiratory Trauma Not classified **Operative admission ** **diagnosis** Cardiovascular Gastrointestinal Genitourinary Metabolic/Endocrine Musculoskeletal/fSkin Neurologic Respiratory Transplant Trauma Not classified	187(6.33) 292(9.88) 168(5.69) 29(0.98) 95(3.21) 45(1.52) 424(14.45) 1015(34.35) 121(4.09) 4(0.14) 57(1.93) 135(4.57) 69(2.34) 5(0.17) 36(1.22) 240(8.12) 15(0.51) 3(0.10) 11(0.37) 4(0.14)	177(6.35) 275(9.87) 158(5.67) 27(0.97) 87(3.12) 39(1.40) 404(14.59) 951(34.12) 111(3.98) 4(0.14) 55(1.97) 129(4.63) 68(2.44) 5(0.18) 34(1.22) 231(8.29) 15(0.54) 3(0.11) 10(0.36) 4(0.14)	10(5.95) 17(10.12) 10(5.95) 2(1.19) 8(4.76) 4(2.42) 20(11.90) 64(38.10) 10(5.95) 0(0) 2(1.19) 6(3.57) 1(0.60) 0(0.0) 2(1.19) 9(5.36) 0(0.0) 0(0.0) 1(0.60) 0(0)	0.573
**Organ support during 24 ** **hours of index ICU admission)** Invasive ventilation Non-invasive ventilation Cardiovascular support Therapeutic Antibiotics use Renal replacement therapy	495(16.75) 348(11.78) 369/2929(12.60) 2236/2929(76.34) 89/2569(3.46)	465(16.68) 322(11.55) 340/2764(12.30) 2101/2764(76.01) 78/2422(3.22)	30(17.86) 26(15.48) 29/165(17.58) 135/165(81.82) 11/147(7.48) *	0.693 0.126 0.047 * 0.088 0.006 *
Use of MV>24h during ICU admission	738(24.97)	689(24.72)	49(29.17)	0.196
Duration of MV in days, median (Q1-Q3) Ventilation free days, median (Q1-Q3) Duration of antibiotic therapy in days, median (Q1-Q3)) Duration of cardiovascular support in days, median (Q1-Q3) Duration of RRT in days, median (Q1-Q3)	2(1-5) 2(1-4) 3(1-5) 2(1-3) 2(1-4)	2(1-5) 2(1-4) 3(1-5) 2(1-3) 2(1-4)	2(1-5) 2(1-4.5) 2(1-3) 3(1-5)	0.1603 0.9012 0.0366 * 0.0843 0.0152 *
APACHE II score, median (Q1-Q3)	9(5-15)	8(4-12)	8.5(14-5)	0.0364 *
Duration from discharge to readmission median (Q1-Q3)) , n=161			3(1-8)	
Index ICU stay; Length of stay in days, median (Q1-Q3) mean(SD)	3(2-6) 4.80(6.91)	3(2-6) 4.77(6.97)	3(2-6) 5.18(5.92)	0.2249
**Night vs daytime discharge** Night Day	932(31.54) 2023(68.46)	883(31.68) 1904(68.32)	49(29.17) 119(70.83)	0.495
**Weekend discharge** Yes No	421(14.25) 2534(85.75)	394(14.14) 2393(85.86)	27(16.07) 141(83.93)	0.486

*Significant at 95% of level of confidence ^†^ Not applicable

Patients who were readmitted had a higher requirement of renal replacement therapy in the first 24 hours of ICU admission (7.48% vs. 3.22 %; p= 0.006) and a higher ICU mortality rate compared to the non-readmitted patients (27.98% vs. 21.69 % (p=0.043) ( Table 1 and Table 2). The ICU mortality of patients readmitted following night time ICU discharge was higher compared to those discharged during the day (38.46 vs 21.36 %, p= 0.016). Observed ICU mortality rate in early vs. late readmitted patients was comparable (31.34 vs 25.75 %, p= 0.428). Among the ICU patients with available hospital mortality data (N=67), the in-hospital mortality rate was 70.15% (N=47).

**Table 2.** ICU outcomes and characteristics of readmission.

Characteristic	Outcome at ICU discharge			
	Length of ICU stay days, median (Q1, Q3)	Alive	Dead	P value
Readmission Night time n=65(38.69%) Day time n=103(61.31%)	3(1,6) 3(2,6)	40(61.54) 81(78.64)	25(38.46) 22(21.36)	0.016 *
Early readmission n=67 (39.88%) Late readmission n= 101(60.12%)	1(1,0) 7(11,4)	46(68.66) 75(74.26)	21(31.34) 26(25.74)	0.428
Readmitted to ICU Yes (n=168) No (n=3559)		121(72.02) 2787(78.31)	47(27.98) 772(21.69)	0.043 *

*Significant at 95% of level of confidence

Independent factors associated with ICU readmission were age, nonoperative route of admission, RRT during the first 24 hours of index ICU admission, cardiovascular support during the first 24 hours of index ICU admission, duration of antibiotic therapy, duration of RRT, and APACHE II score ( Table 1). In the multivariate analysis, age, non-operative diagnosis, cardiovascular support during the first 24 hours and RRT during the first 24 hours were associated with readmission ( Table 3). The area under the receiver operating characteristic curve of the multivariable model was 0.61.

**Table 3.** Univariate and multivariate logistic regression analysis addressing risk factors for intensive care unit readmission (n = 2919 patients).

Characteristics	Univariate analysis		Multivariate analysis	
	OR(95% CI)	p-Value	OR(95% CI)	p-Value
Age ^$^	1.01(1.001-1.018)	0.024 *	1.009(0.999-1.019)	0.047
Male gender	0.801(0.584-1.099)	0.268		
Non-operative admission ^$^	1.(1.101-2.800)	0.018 *	1.772(0.986-3.184)	0.038
**Comorbidities ^$^** Hypertension Diabetes Type 2 Diabetes Cardiovascular diseases Hypothyroidism Renal failure(moderate to severe) Chronic pulmonary disease Respiratory disease	1.144(0.641-2.042) 0.849(0.343-2.101) 1.062(0.398-2.832) 0.961(0.327-2.823) 2.458(1.021-5.920) 1.826(0.609-5.477) 1.767(0.702-4.448) 0.913(0.118-7.059)	0.648 0.724 0.905 0.942 0.245 0.089 0.227 0.930		
**Organ support during first 24 hours** Invasive ventilation ^$^ Non-invasive ventilation ^$^ Cardiovascular support ^$^ Use of antibiotic ^$^ Renal replacement therapy ^$^	1.086(0.772-1.631) 1.401(0.908-2.163) 1.520(1.002-2.306) 1.420(0.947-2.129) 2.699(1.307-5.573)	0.693 0.327 0.049 * 0.090 * 0.007 *	1.704(1.015-2.862) 2.268(1.080-4.764)	0.044 0.031
Use of MV > 24h during index ICU admission	1.253(0.889-1.768)	0.197		
Length of index ICU stay	1.007(0.991-1.024)	0.405		
Night discharge of index ICU admission ^$^	0.888(0.631-1.250)	0.496		
Weekend discharge of index iCU admission ^$^	1.163(0.760-1.779)	0.486		
Duration of MV of index ICU admission	0.997(0.967-1.029)	0.873		
Ventilation free days of index ICU admission	1.021(0.974-1.071)	0.391		
Duration of antibiotic therapy of index ICU admission ^$^	1.015(0.991-1.040)	0.126 *		
Duration of cardiovascular support of index ICu admission	1.014(0.957-1.076)	0.631		

^*^ Significant with 80% level of confidence ^$^ Variable included in multivariate regression analysis

## Discussion

This study provides new evidence on the incidence, characteristics and impact of ICU readmission from the tertiary hospitals in Kathmandu, Nepal. The study leveraged data from a recently implemented multicenter electronic ICU registry capturing case mix, processes and clinical outcomes alongside organisational factors associated with service delivery. The ICU readmission incidence was 5.69 %, and is comparable to other studies reported from healthcare settings internationally, where ICU readmission rates range from 2.5 to 10% ^4, 10, 11, 25^. ICU mortality and morbidity appears to be higher in this population compared to international cohorts (27 % vs 21%) ^25^. The utilisation of ICU resources including mechanical ventilation, and invasive therapies including renal replacement therapy (RRT) in ICU was higher during the reradmission epiodes, than for index admissions. The identified factors associated identifies with ICU readmission were age, non-operative admissions, the use of renal and cardiovascular support within the first 24 hours of index admission. These findings are consistent with other studies conducted internationally including in other resource constrained health care systems, whereby older comorbid patients had higher severities of illness at admission, and were more likely to require ICU readmission ^26, 27^.

Nighttime discharge following index admission was associated with increased ICU mortality compared to day time discharge for readmitted patients (37.5 vs 20.79 %). Similar findings have been found elsewhere ^28– 30^. We did not explore the impact on discharge timing for all ICU patients in this study given the limited availability of hospital outcomes. Lower staffing ratios, absence of senior staff on the wards at night time inadequate clinical handover have been associated with risk of readmission elsewhere ^30– 32^. Our study could not find any difference in ICU outcome for readmitted patients based on timing of readmission. Internationally reports of association vary, however definitions of early versus late are inconsistent ^3, 26, 33^. The findings of this study provide preliminary data on burden, risk and impact of ICU readmission. The identification of modifiable risk factors associated with ICU readmission is a key finding to inform subsequent improvement interventions to reduce the rate of avoidable readmission.

Whilst providing new data on readmission, this study was limited by its retrospective nature, limited hospital outcome data and non representation of hospitals outside of Kathmandu. However this initial evaluation of existing care using routinely available service data is an important step in identifying the prevalence, case mix and characteristics of readmission in the Nepal ICU population. Furthermore the mortality associated with ICU readmission is an important finding. Organisational and patient characteristics identified here are currently being used to inform the development of risk prediction model for readmission and will inform design of future interventions related to discharge planning. Once developed, external validation of this risk prediction model will be sought from other Asia registries in CCAA.

## Conclusions

The results from this study show that the participating ICUs in this study have comparable incidence and patient risk factors for ICU readmission to other international studies. However outcomes are poorer, and care processes associated with risk of readmission may be different compared to more resource rich settings.

## Data Availability

Figshare: Incidence, risk and impact of ICU readmission on patient outcomes and resource utilisation in tertiary level ICUs in Nepal, https://doi.org/10.6084/m9.figshare.20726392 ^17^. This project contains the following underlying data: De-identified_NICR_patient_data_29_08_2022.xlsx Core_variables_definition.pdf Additional file 1_Characteristics of all ICU patients.docx Data are available under the terms of the Creative Commons Attribution 4.0 International license (CC-BY 4.0).
