# Supplementary material for: Incidence, risk and impact of ICU readmission on patient outcomes and resource utilisation in tertiary level ICUs in Nepal: A cohort study
Source: Wellcome Open Res. 2025 Feb 24;7:272. Originally published 2022 Nov 3. [Version 3] doi: 10.12688/wellcomeopenres.18381.3 (PMC11959250; doi:10.12688/wellcomeopenres.18381.3)
Supplement: Peer review history

# Reviewer report: Reviewer response for version 3

**DOI:** 10.21956/wellcomeopenres.26310.r121281

**Published:** 2025-03-31

**Authors:** Younsuck Koh

**Affiliations:** 1 Department of Medicine, Asan Medical Center, University of Ulsan College of Medicine, Seoul, South Korea

**General comments:**

 The authors aim to find the determinants of risk attributable to ICU readmission under low ICU resource clinical settings in Kathmandu, Nepal. The authors found that the readmitted patients used more ICU resources and showed higher mortality rate than other cases.

**Major comments:**

1. It would be better to present the four ICUs resource settings of the tertiary hospitals.
2. Did the frequency of night time discharges differ among the four ICUs? If so, were ICU resource settings, including critical care staffs, associated with the night discharge rate?
3. The median duration of mechanical ventilation support for the subjects was 2 days, and worsening respiratory failure, a common reason for ICU readmission in other study, was not reason in this study. Can authors explain the difference?
4. What was the proportion of patients discharged from ICU receiving vasopressors?
5. Patients who were readmitted had a higher requirement of RRT in the first day of ICU admission, and the duration of RRT use was longer in the readmission group. What proportion of patients who were readmitted to ICU after being RRT require RRT again? If the rate is high, the need for a guideline of RRT discontinuation should be suggested in the discussion.

Is the work clearly and accurately presented and does it cite the current literature?

Partly

If applicable, is the statistical analysis and its interpretation appropriate?

Yes

Are all the source data underlying the results available to ensure full reproducibility?

Yes

Is the study design appropriate and is the work technically sound?

Yes

Are the conclusions drawn adequately supported by the results?

Partly

Are sufficient details of methods and analysis provided to allow replication by others?

Yes

Reviewer Expertise:

Critical Care Medicine

I confirm that I have read this submission and believe that I have an appropriate level of expertise to confirm that it is of an acceptable scientific standard, however I have significant reservations, as outlined above.

# Reviewer report: Reviewer response for version 3

**DOI:** 10.21956/wellcomeopenres.26310.r119380

**Published:** 2025-03-16

**Authors:** Adam Laytin

**Affiliations:** 1 Johns Hopkins University, Baltimore, Maryland, USA

Overall the manuscript is clear and well written, reporting on an important topic for critical care systems strengthening in resource-limited settings. There are a few minor issues where results could be presented more clearly.

 - In methods, you define weekend as "seven pm Friday to seven am Sunday"--does Nepal observe a one day weekend, or should the weekend end on Monday morning?

 - In results, you report "The proportion of readmitted patients was significantly higher among non-operative patients compared to post-operative patients (87.50% vs. 12.50%, p=0.016)"--those percentages aren't meaningful without knowing breakdown of non-op vs post-op patients--it would make more sense to report % of non-op pts readmitted vs % of post-op pts readmitted

 - Similarly, % of readmissions who were downgraded on nights and weekends is difficult to interpret without knowing what percentage of patients were downgraded on nights and weekends. It would make more sense to report % of patients downgraded on nights and weekends who were readmitted in comparison to weekday daytime downgrades

 - Table 1 seems to have a few errors ("Duration of antibiotic therapy" has an empty cell, "median APACHE II" is lower for both subgroups than the overall sample and one IQR doesn't make sense)

 - Table 2 is confusing and would benefit from a legend clarifying what it shows. In particular, "Readmission night time vs day time" is confusing--are you talking about readmission for patients downgraded at night or day or about patients readmitted during night vs day? Is this table showing something different than the bottom of table 1?

 - Table 3 "Organ support during first 24 hours" rows don't line up

 - In discussion, this sentence is confusing and does not seem to align with results section--"Nighttime discharge following index admission was associated with increased ICU mortality compared to day time discharge for readmitted patients (37.5 vs 20.79 %)." In the results you report "The ICU mortality of patients readmitted following night time ICU discharge was higher compared to those discharged during the day (38.46 vs 21.36 %, p= 0.016)." In contrast, table 1 seems to suggest no increased frequency of ICU readmission among patients night/weekend downgrades. I don't know that you need to restate the findings in the discussion, but reporting should be clear and consistent throughout.

 - In discussion, you mention "The identification of modifiable risk factors associated with ICU readmission is a key finding to inform subsequent improvement interventions to reduce the rate of avoidable readmission." What improvement interventions are informed by the findings of this study?

 - The last paragraph of discussion mentions CCAA, which is not referenced anywhere else in the manuscript. Either elaborate or remove.

Is the work clearly and accurately presented and does it cite the current literature?

Partly

If applicable, is the statistical analysis and its interpretation appropriate?

Partly

Are all the source data underlying the results available to ensure full reproducibility?

Yes

Is the study design appropriate and is the work technically sound?

Yes

Are the conclusions drawn adequately supported by the results?

Yes

Are sufficient details of methods and analysis provided to allow replication by others?

Yes

Reviewer Expertise:

Epidemiology, QI, trauma, critical care, ICU processes of care and outcomes

I confirm that I have read this submission and believe that I have an appropriate level of expertise to confirm that it is of an acceptable scientific standard, however I have significant reservations, as outlined above.

# Reviewer report: Reviewer response for version 2

**DOI:** 10.21956/wellcomeopenres.21165.r61772

**Published:** 2023-07-04

**Authors:** Adam Laytin

**Affiliations:** 1 Johns Hopkins University, Baltimore, Maryland, USA

Abstract:

- Well structured and clear. "eCRF" is not defined where used here or in abbreviation list. It's also not used in the methods section. Either eliminate it here or elaborate in methods section.

Introduction:

- Clear, concise, appropriate for study question.

Methods:

- Overall clear, fairly concise, appropriate.
- The abstract mentions increased mortality in readmitted patients who were discharged at night. However, investigation for risk factors for mortality in readmitted patients (i.e. failure to rescue) isn't described in methods and is only touched on in results. It would make sense to either include that analysis in Table 2 or omit it all together. 
- NICRF should be defined the first time it is used.
- Authors mention comparison between weekday and weekend admissions in the second paragraph as a secondary outcome but not in the third paragraph as a comparison that was actually performed.
- Variables such as hospital LOS and hospital mortality that are not presented in results should not be included in methods

Results:

- Be consistent with "medical vs surgical" admissions or "non-op vs post-op".
- "The patients with medical diagnoses at index admission had a higher ICU readmission rate compared to the surgical patients (87.50% vs. 12.50%, p=0.016)"--those percentages don't make sense with an overall 5.69% ICU readmission rate. It looks like the authors are presenting percentage of readmitted patients who were surgical, not percentage of surgical patients who were readmitted.
- Weekend readmission rates are not mentioned in the text.
- Table 1"Duration from discharge to readmission" does not need to be presented as it is already included in text. A p value should be reported for each of the admission diagnoses.
- "Patients who were readmitted had... higher ICU mortality rate compared to the non-readmitted patients" is a very important but very confusing statement. Are you saying that the post-ICU discharge mortality was 21.69% for patients who were not readmitted, or that the ICU mortality on index admission was 21.69%? From the methods, it sounded like you don't have hospital mortality data for patients who did not have ICU readmission, but that would be useful to report if available.
- Table 2 is confusing. It seems like it should present the bivariable investigation for risk factors for increased mortality in patients with ICU readmission given that this is included for early vs late and day vs night readmissions. The "readmitted to ICU" row does not fit with the formatting of the table and is already addressed in the text--it can be excluded here. Why are 3359 patients without ICU readmission reported here, instead of 2787 as reported in table 1?
- Table 3 "Organ support during first 24 hours" appears to have a formatting issue and rows do not align. It would make more sense to put this table after table 1, since it also addresses risk factors for ICU readmission (vs outcomes/characteristics of readmission). 
- I would recommend against reporting hospital mortality given the low n and high risk of biased reporting.

Discussion:

- Concise with review of relevant related literature.
- Would be helpful to address whether night time admissions in this setting reflect patients being bumped out of the ICU before they are ready to make room for sicker patients.
- What are implications for QI, beyond developing a risk prediction model?

Is the work clearly and accurately presented and does it cite the current literature?

Partly

If applicable, is the statistical analysis and its interpretation appropriate?

Partly

Are all the source data underlying the results available to ensure full reproducibility?

Yes

Is the study design appropriate and is the work technically sound?

Yes

Are the conclusions drawn adequately supported by the results?

Yes

Are sufficient details of methods and analysis provided to allow replication by others?

Yes

Reviewer Expertise:

Epidemiology, QI, trauma, critical care, ICU processes of care and outcomes

I confirm that I have read this submission and believe that I have an appropriate level of expertise to confirm that it is of an acceptable scientific standard, however I have significant reservations, as outlined above.

# Response

**Published:** 2025-02-19

**Authors:** Diptesh Aryal

**Affiliations:** Nepal Intensive Care Research Foundation, Nepal

Dear Reviewer, Thank you for your patience and for the opportunity to revise our manuscript. We sincerely apologize for the delayed response, as other personal commitments required our attention. We appreciate the encouraging, critical and constructive comments on this manuscript. The comments have been very thorough and useful in improving the manuscript. We strongly believe that the comments and suggestions have increased the scientific value of the revised manuscript by many folds. We have taken them fully into account in revision. We are submitting the corrected manuscript with the suggestion to incorporate the manuscript in the track change mode. Our responses to all the comments are as follows: Abstract:

- Well structured and clear. "eCRF" is not defined where used here or in the abbreviation list. It's also not used in the methods section. Either eliminate it here or elaborate in the methods section.
- **Response**: The term eCRF has been removed from abstract.

Methods:

- The abstract mentions increased mortality in readmitted patients who were discharged at night. However, investigation for risk factors for mortality in readmitted patients (i.e. failure to rescue) isn't described in methods and is only touched on in results. It would make sense to either include that analysis in Table 2 or omit it all together.
- **Response**: Thank you for your valuable comment. We would like to clarify that "failure to rescue" is not mentioned in the manuscript. The investigation of risk factors for mortality in readmitted patients was not a focus of this study. The mention in the abstract regarding increased mortality in readmitted patients who were discharged at night was intended to be a descriptive finding rather than an analysis of specific risk factors for mortality.
- NICRF should be defined the first time it is used.
-  
**Response**: "Nepal Intensive Care Research Foundation (NICRF)" is now defined where it is first used.
- Authors mention comparison between weekday and weekend admissions in the second paragraph as a secondary outcome but not in the third paragraph as a comparison that was actually performed.
- **Response:** Thank you for your feedback. We have revised the sentence to clearly state the comparisons made in the study, including the comparison between weekday and weekend admissions:
- Comparisons were made between patients with and without readmission to the ICU, between readmissions occurring at night versus daytime, between early versus late readmissions, and between weekday versus weekend readmissions.
- Variables such as hospital LOS and hospital mortality that are not presented in results should not be included in methods
- **Response: **Thank you for your comment. Hospital length of stay (LOS) has been removed from methods section. We would like to clarify that hospital mortality is presented in the results section in text.

Results:

- Be consistent with "medical vs surgical" admissions or "non-op vs post-op".
- Thank you for your feedback. "medical vs surgical"  has been replaced with "non-operative patients and post-operative" for clarity and consistency.
- "The patients with medical diagnoses at index admission had a higher ICU readmission rate compared to the surgical patients (87.50% vs. 12.50%, p=0.016)"--those percentages don't make sense with an overall 5.69% ICU readmission rate. It looks like the authors are presenting the percentage of readmitted patients who were surgical, not percentage of surgical patients who were readmitted.
- **Response:** I agree with your suggestion. The sentence has been rephrased.

Weekend readmission rates are not mentioned in the text.

- **Response: **Thank you for your feedback. We have added the comparison of weekend and weekday readmission rates to the text and included this information in Table 1.
- Table 1"Duration from discharge to readmission" does not need to be presented as it is already included in text. A p value should be reported for each of the admission diagnoses.

- **Response:** Changes made as suggested.
- "Patients who were readmitted had... higher ICU mortality rate compared to the non-readmitted patients" is a very important but very confusing statement. Are you saying that the post-ICU discharge mortality was 21.69% for patients who were not readmitted, or that the ICU mortality on index admission was 21.69%? From the methods, it sounded like you don't have hospital mortality data for patients who did not have ICU readmission, but that would be useful to report if available.
- **Response:** Readmitted patients had a significantly higher mortality rate compared to the ICU mortality on index admission (27.98% vs. 21.69 % (p=0.043). The hospital mortality data have been removed.
- Table 2 is confusing. It seems like it should present the bivariable investigation for risk factors for increased mortality in patients with ICU readmission given that this is included for early vs late and day vs night readmissions. The "readmitted to ICU" row does not fit with the formatting of the table and is already addressed in the text--it can be excluded here. Why are 3359 patients without ICU readmission reported here, instead of 2787 as reported in table 1?
- **Response **: The "readmitted to ICU" row has been removed.
- Table 3 "Organ support during first 24 hours" appears to have a formatting issue and rows do not align. It would make more sense to put this table after table 1, since it also addresses risk factors for ICU readmission (vs outcomes/characteristics of readmission).
- **Response:** Corrected.
- I would recommend against reporting hospital mortality given the low  and high risk of biased reporting.
- **Response:** Hospital mortality has been removed.

  Discussion:

- Concise with review of relevant related literature.
- **Response: **We appreciate the reviewer's feedback. The discussion already integrates key findings from relevant studies; however, we will ensure that the literature review remains focused and directly supports our study’s objectives without unnecessary elaboration.
- Would be helpful to address whether night time admissions in this setting reflect patients being bumped out of the ICU before they are ready to make room for sicker patients.
- **Response: **We acknowledge this possibility and we have included a discussion on whether nighttime ICU discharges could reflect premature transfers due to ICU bed availability constraints.
- What are implications for QI, beyond developing a risk prediction model?
- **Response:** We have added a sentence about the possibility of designing an intervention to reduce adverse events associated with early ICU readmission.

# Reviewer report: Reviewer response for version 2

**DOI:** 10.21956/wellcomeopenres.21165.r55316

**Published:** 2023-03-01

**Authors:** David Pilcher

**Affiliations:** 1 Department of Intensive Care, Alfred Hospital, Melbourne, Vic, Australia

I commend the authors on their revisions and enjoyed reading the paper. Well done and thank you.

Is the work clearly and accurately presented and does it cite the current literature?

Partly

If applicable, is the statistical analysis and its interpretation appropriate?

Partly

Are all the source data underlying the results available to ensure full reproducibility?

Yes

Is the study design appropriate and is the work technically sound?

Yes

Are the conclusions drawn adequately supported by the results?

Yes

Are sufficient details of methods and analysis provided to allow replication by others?

No

Reviewer Expertise:

Intensive Care

I confirm that I have read this submission and believe that I have an appropriate level of expertise to confirm that it is of an acceptable scientific standard.

# Reviewer report: Reviewer response for version 1

**DOI:** 10.21956/wellcomeopenres.20375.r53178

**Published:** 2022-12-07

**Authors:** David Pilcher

**Affiliations:** 1 Department of Intensive Care, Alfred Hospital, Melbourne, Vic, Australia

Thank you for asking me to review this paper which describes the characteristics and outcomes of -patients readmitted to 4 ICUs in Kathmandu Nepal between Sept 2019 and Feb 2021. The authors report a readmission proportion of 5.6%, identify risk factors for readmission and highlight discharge at night as risk factor for mortality. The paper is generally well written, an important topic, highlights the use of registry data and provides locally valuable information to influence clinical practice.

 I have a number of comments that should be considered by the authors.

 The authors state their paper is about ‘unplanned readmissions’ to ICU. Does this mean there were planned readmissions (e.g. elective surgical) to ICU that were not included in the study? This should be clarified or are they including all readmission episodes for all patients? This should be more explicitly explained or they should remove the word ‘unplanned’.

 The Intro rightly highlights patient and organisational risk factors for readmission.

 Why were burns patients excluded? I see no rationale for excluding them.

 The authors should be clearer about the outcomes they are examining.

 ICU length of stay (LOS) is listed as outcome. Does this refer to the original LOS for all patients (if so it should not be considered as an outcome of the study since this occurs before the readmission episode, or dose this refer to the LOS of the readmission episode? If the former it is still important to report and should be considered as a potential predictor of readmission.

 ‘Mortality’ is stated as an outcome. Is this in ICU or in hospital. Results and text later imply ‘in-ICU’. This needs clarification. It would be highly preferable to report both. In a study of this size and given this is a paper inherently about the interface between ICU and ward, hospital mortality should ideally be available and reported. If not available this should be discussed as a limitation.

 Primary outcome of the study appears to be ‘readmission’ – should be explicitly stated as such. The other outcomes are secondary.

 APACHE diagnostic grouping are reported but no overall severity of illness score. It is likely that acute illness severity also influences readmission risk. Why has this been left out?

 Figure 1 –

- This figure is extremely difficult to understand and should be made much clearer.
- What are ‘duplicates’? Are these duplicated records for the same patient’s ICU admission. If so these should be excluded up front in any data cleaning exercise and potentially don’t even need inclusion in the flow chart.
- Presumably Diseased = Deceased?
- List number of admissions & number of patients.
- Some numbers in the boxes on the left don’t make sense (4346 – 170 is not 4006).
- Index admission for readmitted patients deceased at ICU discharge – What does this mean?
- There are 54 patient excluded for ‘other (unclassified reasons)’. In a study of this size, this could be important. Why are these patients excluded from a study like this?

Table 1 –

- My preference would be to provide exact P values for all variables using comparative statistics here.
- ICU outcome – death? In ICU or at any time in hospital? See earlier comment also.
- LOS in ICU - Is this for the first admission? What was the LOS for the readmission episodes of those who cam back to ICU? How many patients had more than one readmission episode?
- If considering resource implication of readmissions, then it would be appropriate to include mean LOS values in addition to median & IQR.
- I was surprised to see duration of ventilation reported as mean SD. This typically has a non-parametric distribution in most studies. Please check.
- Separate outcomes section of table from potential ‘exposure variables’ which might influence the chance of readmission (i.e. put night discharge and weekend dc elsewhere)

Table 2

- I think this should be pivoted around so that it matches format of table 1. There is probably no need to report alive and dead as separate columns.

The paper mixes in night discharge from the index admissions as a risk factor for mortality of these patients. If the purpose of the paper is to describe and identify the risk factors for readmission, then reporting whether night discharge is a risk factor for mortality is of secondary importance and less relevance to this study. In addition, if the mortality outcome the authors report is in-ICU mortality only, then reporting risk factors for mortality in the readmitted patients is may be confounded or biased by lack of mortality outcomes in the other patients who were discharged from ICU. It is quite possible that night discharge is a RF for mortality in all patients not just in patients readmitted to ICU. I recommend removing or reducing the focus on this.

 The whole paper should have a proof read to provide more clarity when the authors are referring to ICU admission episodes, ICU readmission episode or patients. It is difficult to follow sometimes when authors are referring to characteristics of the patient as opposed to characteristics of the ICU episode. E.g.

*“…and invasive therapies including renal replacement therapy (RRT) in ICU was higher in patients readmitted, than for index admissions.”*

 Do the authors mean that patients who were readmitted had greater RRT use during their initial admission to ICU than during the same patient’s subsequent readmission episode, or that patients who were readmitted to ICU had higher use of RRT during their initial ICU episode than patients who were not readmitted to ICU?

 The authors state their intention to create a readmission risk prediction model. Most readmission models reported have low discriminatory power. They tend to perform better if additional outcomes such as death after ICU discharge. What was the AUC of their multivariable model? If not that high, then this also highlights the difficulty in creating such models in future. However, it does not distract from the clinical relevance of their findings which identify independent risk factors for readmission (as the authors rightly highlight in the Discussion).

 Hospital level process of care - where any measured? Many of these are mentioned in the Discussion.

- Liaison service
- Handover processes
- Routine post-ICU review
- Insurance/payer status
- Nursing ratios in ICU or in ward
- Public vs. private (understanding that reporting of information which potentially might identify the hospitals may not be possible)

Conclusion

 Although statements such as “ *…The utilisation of finite and expensive resources (organ support, ICU bed days) is higher in this population compared to HICs.*” may be ok to include and consider in the Discussion, the inclusion of this statement in the Conclusion is not justified without presentation of data. It should be either removed or backed up with information to support. I see no evidence that the relative resource utilisation of patients readmitted to ICU is any different in this study to the HIC where I work and was in fact surprised to see how similar it was.

Is the work clearly and accurately presented and does it cite the current literature?

Partly

If applicable, is the statistical analysis and its interpretation appropriate?

Partly

Are all the source data underlying the results available to ensure full reproducibility?

Yes

Is the study design appropriate and is the work technically sound?

Yes

Are the conclusions drawn adequately supported by the results?

Yes

Are sufficient details of methods and analysis provided to allow replication by others?

No

Reviewer Expertise:

Intensive Care

I confirm that I have read this submission and believe that I have an appropriate level of expertise to confirm that it is of an acceptable scientific standard, however I have significant reservations, as outlined above.

# Response

**Published:** 2023-02-21

**Authors:** Diptesh Aryal

**Affiliations:** Nepal Intensive Care Research Foundation, Nepal

Dear David Pilcher,

 Thank you for taking the time to review our paper "Incidence, risk and impact of ICU readmission on patient outcomes and resource utilisation in tertiary level ICUs in Nepal" and for providing your valuable feedback. We appreciate your thoughtful comments and suggestions, which have helped us to improve the quality of our work. We have carefully considered your comments and have made revisions to address your concerns. Your input has been instrumental in helping us to refine our paper, and we are grateful for your contributions. Best Regards Diptesh Aryal

**Reviewer's Comments and Responses:**

 The authors state their paper is about ‘unplanned readmissions’ to ICU. Does this mean there were planned readmissions (e.g. elective surgical) to ICU that were not included in the study? This should be clarified or are they including all readmission episodes for all patients? This should be more explicitly explained or they should remove the word ‘unplanned’.  

*Thank you. We included all the readmission episodes for all the patients. We have removed “unplanned” in the revised manuscript. In our settings, readmissions that are part of the usual plan typically only occur in specific situations, such as following a two-stage surgical operation, and they are documented as separate admission episodes.*   

 The Intro rightly highlights patient and organisational risk factors for readmission. Why were burns patients excluded? I see no rationale for excluding them.

*Thank you for your comment. We have included burn patients in the analysis and updated the results.*   

 The authors should be clearer about the outcomes they are examining. ICU length of stay (LOS) is listed as outcome. Does this refer to the original LOS for all patients (if so it should not be considered as an outcome of the study since this occurs before the readmission episode, or dose this refer to the LOS of the readmission episode? If the former it is still important to report and should be considered as a potential predictor of readmission.

*Thank you for the comment. We have clarified this in the revised manuscript. *  

 ‘Mortality’ is stated as an outcome. Is this in-ICU or in-hospital. Results and text later imply ‘in-ICU’. This needs clarification. It would be highly preferable to report both. In a study of this size and given this is a paper inherently about the interface between ICU and ward, hospital mortality should ideally be available and reported. If not available this should be discussed as a limitation.

*Thank you for this feedback. We have clarified the outcomes in the results and discussion. We have included hospital outcomes (where available) and noted this as a limitation in the revised manuscript.*

 Primary outcome of the study appears to be ‘readmission’ – should be explicitly stated as such. The other outcomes are secondary.  

*Thank you for your comments. We have made the changes in the revised manuscript.*

 APACHE diagnostic grouping is reported but no overall severity of illness score. It is likely that acute illness severity also influences readmission risk. Why has this been left out?  

*We have added  APACHE II illness severity to the  revised manuscript.*

 Figure 1 –

- This figure is extremely difficult to understand and should be made much clearer
*We have updated the figure 1.  *
- What are ‘duplicates’? Are these duplicated records for the same patient’s ICU admission. If so these should be excluded up front in any data cleaning exercise and potentially don’t even need inclusion in the flow chart.
*Removed*.   
- Presumably Diseased = Deceased?
*Corrected. *  
- List number of admissions & number of patients. 
*Added. *
- Some numbers in the boxes on the left don’t make sense (4346 – 170 is not 4006). 
*This has now been revised and changed in the manuscript.*
*  *  
- Index admission for readmitted patients deceased at ICU discharge – What does this mean?  
*Patients deceased following ICU index admission, were removed since they cannot be readmitted.*
- There are 54 patients excluded for ‘other (unclassified reasons)’. In a study of this size, this could be important. Why are these patients excluded from a study like this?  
*These patients were excluded because they were either discharged alive directly from the ICU to either home or another healthcare facility for end of life care. Corrected in fig 1. *  

Table 1 –

- My preference would be to provide exact P values for all variables using comparative statistics here. 
*Added*
- ICU outcome – death? In ICU or at any time in hospital? See earlier comment also.
*Thank you. Clarified as above. *
- LOS in ICU - Is this for the first admission? What was the LOS for the readmission episodes of those who came back to ICU? How many patients had more than one readmission episode? 
*Thank you. Yes LOS in ICU is for the index admissions (first admissions). LOS  for the readmission episodes were reported in table 2. No patients reported with more than one readmission episode.*    
- If considering resource implication of readmissions, then it would be appropriate to include mean LOS values in addition to median & IQR.
*Thank you for the suggestion.* *Mean LOS with SD has been added to table 1. *
- I was surprised to see the duration of ventilation reported as mean SD. This typically has a non-parametric distribution in most studies. Please check. 
*Thank you for highlighting. It has now been corrected in the table. *
- Separate outcomes section of table from potential ‘exposure variables’ which might influence the chance of readmission (i.e. put night discharge and weekend dc elsewhere)  
*Thank you. Table 2 has been revised to address the concern. *  

Table 2

 I think this should be pivoted around so that it matches format of table 1. There is probably no need to report alive and dead as separate columns.   

*Thank you. Table 2 described the readmission episodes not the characteristics of index admission episodes .*

 The paper mixes in night discharge from the index admissions as a risk factor for mortality of these patients. If the purpose of the paper is to describe and identify the risk factors for readmission, then reporting whether night discharge is a risk factor for mortality is of secondary importance and less relevance to this study. In addition, if the mortality outcome the authors report is in-ICU mortality only, then reporting risk factors for mortality in the readmitted patients is may be confounded or biased by lack of mortality outcomes in the other patients who were discharged from ICU. It is quite possible that night discharge is a RF for mortality in all patients not just in patients readmitted to ICU. I recommend removing or reducing the focus on this.  

*Thank you. Table 2 has been revised to address the concern. *  

 The whole paper should have a proof read to provide more clarity when the authors are referring to ICU admission episodes, ICU readmission episode or patients. It is difficult to follow sometimes when authors are referring to characteristics of the patient as opposed to characteristics of the ICU episode. E.g.   “…and invasive therapies including renal replacement therapy (RRT) in ICU was higher in patients readmitted, than for index admissions.”  

*Thank you. The characteristics of the patients and the ICU episodes have been rephrased for more clarity. *  

 Do the authors mean that patients who were readmitted had greater RRT use during their initial admission to ICU than during the same patient’s subsequent readmission episode, or that patients who were readmitted to ICU had higher use of RRT during their initial ICU episode than patients who were not readmitted to ICU?  

*Addressed above. *  

 The authors state their intention to create a readmission risk prediction model. Most readmission models reported have low discriminatory power. They tend to perform better if additional outcomes such as death after ICU discharge. What was the AUC of their multivariable model? If not that high, then this also highlights the difficulty in creating such models in future. However, it does not distract from the clinical relevance of their findings which identify independent risk factors for readmission (as the authors rightly highlight in the Discussion).  

*The AUC is 0.61. This has been added in the manuscript. *  

 Hospital level process of care - where any measured? Many of these are mentioned in the Discussion.

- Liaison service
- Handover processes
- Routine post-ICU review
- Insurance/payer status
- Nursing ratios in ICU or in ward
- Public vs. private (understanding that reporting of information which potentially might identify the hospitals may not be possible)

*We have amended the discussion to reflect the intention to explore these in the prospective study.  We have reported on the incidence of ICU readmission, the risk factors associated with ICU readmission, the characteristics of the patients, the care processes delivered in ICU, organ support,  resource utilisation, timings of ICU discharge and  the ICU  outcomes. We have not measured the hospital level process of care. We aim to measure these in the prospective study.*

 Conclusion Although statements such as “…The utilisation of finite and expensive resources (organ support, ICU bed days) is higher in this population compared to HICs.” may be ok to include and consider in the Discussion, the inclusion of this statement in the Conclusion is not justified without presentation of data. It should be either removed or backed up with information to support. I see no evidence that the relative resource utilisation of patients readmitted to ICU is any different in this study to the HIC where I work and was in fact surprised to see how similar it was.  

*Thank you. We have removed the statement from the conclusion in the revised manuscript.*
